# Comparison of outcomes of the 50-year follow-up of a randomized trial assessed by study questionnaire and by data linkage: The CONCUR study

**DOI:** 10.1177/17407745241259088

**Published:** 2024-06-22

**Authors:** Mohammad Shahbaz, Jane E Harding, Barry Milne, Anthony Walters, Lisa Underwood, Martin von Randow, Lois Xu, Greg D Gamble, for the ANCHOR Study Group

**Affiliations:** 1 Liggins Institute, The University of Auckland, Auckland, New Zealand; 2 Centre of Methods and Policy Application in Social Sciences, The University of Auckland, Auckland, New Zealand

**Keywords:** Self-reported questionnaire, data linkage, follow-up study, agreement, administrative data sets

## Abstract

**Background/Aims:**

Self-reported questionnaires on health status after randomized trials can be time-consuming, costly, and potentially unreliable. Administrative data sets may provide cost-effective, less biased information, but it is uncertain how administrative and self-reported data compare to identify chronic conditions in a New Zealand cohort. This study aimed to determine whether record linkage could replace self-reported questionnaires to identify chronic conditions that were the outcomes of interest for trial follow-up.

**Methods:**

Participants in 50-year follow-up of a randomized trial were asked to complete a questionnaire and to consent to accessing administrative data. The proportion of participants with diabetes, pre-diabetes, hyperlipidaemia, hypertension, mental health disorders, and asthma was calculated using each data source and agreement between data sources assessed.

**Results:**

Participants were aged 49 years (SD = 1, *n* = 424, 50% male). Agreement between questionnaire and administrative data was slight for pre-diabetes (kappa = 0.10), fair for hyperlipidaemia (kappa = 0.27), substantial for diabetes (kappa = 0.65), and moderate for other conditions (all kappa >0.42). Administrative data alone identified two to three times more cases than the questionnaire for all outcomes except hypertension and mental health disorders, where the questionnaire alone identified one to two times more cases than administrative data. Combining all sources increased case detection for all outcomes.

**Conclusions:**

A combination of questionnaire, pharmaceutical, and laboratory data with expert panel review were required to identify participants with chronic conditions of interest in this follow-up of a clinical trial.

## Background/aims

Self-reported questionnaires are one of the most efficient techniques to get data on a range of health outcomes.^1^ However, participants’ ability to recall diagnoses, willingness to supply medical information, or the complexity of the condition itself may all influence the accuracy and reliability of self-reported data.^2,3^ In addition, self-reported questionnaires can be time-consuming and costly, especially for large-scale studies.^4^

Using administrative data sets is a potentially cost-effective method that may reduce recall and ascertainment bias.^5^ Data linkage can be particularly useful for research that requires large sample sizes, comprehensive data on hard-to-reach sub-populations, and which places little burden on participants.^6^ A systematic review of 65 studies with post-trial follow-up reported data linkage via electronic registries was the most cost-effective method for follow-up of participants over a wide range of outcomes.^7^ However, data linkage was not always feasible, especially in countries where accessing national electronic data was difficult due to lack of a specific health ID number.^7^

Furthermore, utilizing administrative data sets for outcome identification may involve challenges, such as outcome misclassification and missing data, potentially leading to bias.^8,9^ A meta-research study comparing 84 randomized trials using routinely collected data for outcome assessment versus 463 traditional clinical trials found that trials that utilize routinely collected data commonly show smaller treatment benefits due to underestimation of the outcome.^8^

Several studies have investigated discrepancies between self-reported questionnaire and routinely collected data, and reported important but variable discrepancy between diagnosis of chronic conditions assessed by a self-reported questionnaire or by administrative data. Some studies found discrepancies between self-reported and administrative data were greater among older adults.^10,11^ Others have reported the discrepancy varied across cohorts and depended on the outcomes measured, for example, ranging from a kappa of 0.09 for chronic renal failure to 0.86 for type 2 diabetes.^12^ Administrative data had a sensitivity higher than 50% for some conditions, while for others, questionnaire data showed higher sensitivity.^12^ Therefore, combining two data sources may increase case ascertainment for all outcomes, reducing the limitations of both sources.^13,14^

While these studies have focussed on population-based cohorts, there is little information on agreement between data sources for chronic conditions as outcomes after randomized trials. We conducted a follow-up study of 50-year-old offspring of mothers who participated in a randomized trial of antenatal corticosteroids to investigate the effects of prenatal corticosteroid exposure on health in adulthood.^15^ Since it was not feasible to conduct in-person assessments due to the geographically dispersed cohort, we assessed study outcomes through a self-reported questionnaire and by record linkage. This study aimed to determine whether record linkage could replace self-reported questionnaires to identify chronic conditions that were the outcomes of interest for trial follow-up.

## Methods

The Auckland Steroid Trial (1969–1974) was a randomized trial of antenatal betamethasone for prevention of neonatal respiratory distress syndrome carried out in New Zealand.^15,16^ Adult children of mothers recruited to the trial were traced and asked to complete a questionnaire. In addition, consent was sought for record linkage to administrative data sets.

### Data sources

The questionnaire included questions about chronic conditions, medical events, and mental health based on the New Zealand Health Survey.^17^ Participants were asked if they had ever been told by a doctor that they had specific diagnoses and what treatment they had received (Supplementary Table 1).

Administrative data sets were nationwide data sets maintained by the New Zealand Ministry of Health, including: (a) The National Minimum data set (implemented in 1993), a collection of public and private hospital discharge information;^18^ (b) The Pharmaceutical Collection, started in 1 July 1992;^19^ and (c) The National Non-Admitted Patient Collection (implemented on 1 July 2006) providing data on outpatient and emergency department activity.^20^ We also accessed additional data sets as follows:

- The Virtual Diabetes Register, which creates an individual level register of people suspected of having diabetes by their use of diabetes-related services.^21^
- Testsafe commenced in early 2010, which contains laboratory test results from community laboratories in the Northern Region Health District.^22^ This region includes Auckland, where all participants were born, and contains 36% of the New Zealand population.^23^
- The Integrated Data Infrastructure,^24^ which holds individually linked de-identified microdata from multiple government agencies (Supplementary Table 2). Using this data set, we identified all individuals born in New Zealand from December 1969 to February 1974 who were alive in July 2023 and used stratified random sampling to select a cohort of 42,400 individuals of the same sex and ethnicity distribution as the study participants.

### Outcome definitions

We used the following criteria to define the conditions of interest based on records in administrative data sets:

Diabetes mellitus:^25^ Any of:

1. Two haemoglobin A1c (HbA1c) ratios ≥50 mmol/mol.
2. Two fasting plasma glucose concentrations ≥7.0 mmol/L.
3. A 2-h plasma glucose concentration on a 75 g oral glucose tolerance test ≥11.1 mmol/L.
4. One HbA1c ratio ≥50 mmol/mol plus one fasting plasma glucose concentration ≥7.0 mmol/L.
5. Records of prescriptions for metformin, insulin or other diabetes medications.
6. Records of attendance at a diabetes clinic or retinal screening for diabetes.
7. Hospital admissions with diagnostic codes for diabetes mellitus.

Pre-diabetes:^25^ Any of:

1. One HbA1c ratio 41–49 mmol/mol.
2. One fasting plasma glucose concentration 6.1–6.9 mmol/L.
3. A 2-hour plasma glucose concentration on a 75 g oral glucose tolerance test, 7.8–11 mmol/L.

Hyperlipidaemia:^26^ Any of:

1. Total cholesterol concentration >5 mmol/L.
2. LDL (low-density lipoprotein) cholesterol concentration >3.4 mmol/L.
3. Triglyceride concentration >2 mmol/L.
4. Records of prescriptions for lipid-lowering medications.
5. Hospital admissions with diagnostic codes for hyperlipidaemia.

High blood pressure: Any of:

1. Records of prescriptions for antihypertensive medications.
2. Hospital admissions with diagnostic codes for high blood pressure.

Mental health disorders: Any of:

1. Records of prescriptions for depression or anxiety medications.
2. Hospital admissions with diagnostic codes for depression or anxiety.

Asthma: Any of:

1. Records of prescriptions for asthma medications.
2. Hospital admissions with diagnostic codes for asthma.

For diabetes, pre-diabetes, combined diabetes or pre-diabetes (total diabetes), high blood pressure, and asthma, if there were discrepancies between the questionnaire and the administrative data, or if there was evidence from only one administrative data set, an expert panel comprising the five clinician members of the study steering group reviewed all records and reached consensus on the diagnosis.

### Statistical analysis

The proportions of participants with the outcome of interest were calculated with the information available in each data source for participants who consented to that data source, using the Wilson score interval to calculate 95% confidence intervals.^27^

We assessed the agreement between questionnaire and administrative data sets, using the questionnaire as the gold standard. Sensitivity, specificity, and the kappa statistic were calculated (all with 95% confidence intervals). Kappa statistics were categorized using the Cohen criteria as 0–0.20 slight, 0.21–0.40 fair, 0.41–0.60 moderate, 0.61–0.80 substantial, and 0.81–1.00 perfect agreement.^28^ SAS (v9.4 SAS Institute Inc., Cary, NC, USA) statistical software was used for data analysis. An online calculator was used to calculate the Wilson score.^27^

We assessed the validity of the questionnaire and administrative data by assessing agreement between these sources, expert panel review, and the virtual diabetes register. We also compared the proportion of participants with the outcomes of interest in the questionnaire data with age-specific prevalences from the New Zealand Health Survey 2021/2022,^17^ and proportion of participants with the outcomes of interest in the combined data sources with age-sex-specific prevalences from the integrated data infrastructure.

## Results

Of the 1218 infants whose mothers participated in the Auckland Steroid Trial,^15^ 301 died, 255 were lost to follow-up, 154 did not respond, 84 declined, and 424 consented to take part in this follow-up study (Table 1). Of the 424 participants, 415 completed a questionnaire, and 379 consented to all administrative data sources. National Health Index numbers were only available for 420 participants. The study participants had a mean age of 49 years (SD = 1), 50% were male, and 71% were born preterm. Ethnicity was prioritized according to the New Zealand Ministry of Health Guidelines^29^ as 23% Māori, 4% Pacific Peoples, 70% European, and 3% Asian, other or missing (Table 2).

**Table 1.** Baseline characteristics of those eligible who did and did not participate.

	Participated, *N* = 424	Did not participate, *N* = 493	Deceased, *N* = 301
Male	212 (50.0%)	292 (59.2%)	173 (57.5%)
Gestational age at entry, weeks, median (fifth, 95th centiles)	33.1 (27.6, 36.0)	33.4 (27.5, 37.0)	29.6 (24.0, 36.0)
Gestational age at delivery, weeks, median (fifth, 95th centiles)	35.0 (29.3, 40.6)	36.0 (30.7, 41.0)	31.3 (24.0, 40.0)
Multiple pregnancy			
Singleton	370 (87.3%)	437 (88.6%)	261 (86.7%)
Multiple	54 (12.7%)	56 (11.4%)	40 (13.3%)
Term delivery	123 (29.0%)	198 (40.2%)	34 (11.3%)
Birthweight (g) mean (SD)	2317 (747)	2483 (717)	1659 (833)
Birthweight *Z*-score, mean (SD)	−0.36 (0.98)	−0.42 (0.96)	−0.31 (1.28)
5-min Apgar score <7	53 (12.6%)	59 (12.1%)	92 (46.0%)
Respiratory distress syndrome	37 (8.7%)	30 (6.1%)	75 (24.9%)

**Table 2.** Self-reported characteristics of participants.

	*N* = 424 (%)
Prioritized ethnicity	
Māori	97 (23%)
European	296 (70%)
Pacific	20 (4%)
Asian, other, and no response	11 (3%)
Age at follow-up (years), mean (SD)	49.3 (1.0)
Smoking status	
Non-smoker	358 (84%)
Currently smokes	56 (13%)
Tertiary qualification	
None	132 (31%)
Bachelor and national certificate 1–6	182 (43%)
Postgraduate degree (Master, Honours, Certificate)	54 (12%)
PhD	5 (1%)
Other	24 (5%)
Don’t know	18 (4%)
No response	9 (2%)
Employment status	
Working in paid employment	339 (80%)
Not in paid work	33 (8%)
Retired, homemaker, caregiver	13 (3%)
Other	24 (6%)
Unknown	14 (3%)
Self-reported general health	
Excellent	66 (15%)
Very good	141 (33%)
Good	146 (34%)
Fair	49 (11%)
Poor	12 (3%)
No response	10 (3%)
Data availability	
NHI available	420 (99%)
Consent to Testsafe data set	400 (94%)
Consent to pharmaceutical collection	401 (94%)
Consent to NMDS	411 (97%)
Consent to NNPAC	404 (95%)
Out of New Zealand	43 (10%)
Out of northern regional geographic region	158 (37%)

NHI: National Health Index; NMDS: National Minimum Data Set; NNPAC: National Non-Admitted Patient Collection.

The proportion of participants who self-reported a chronic health condition ranged from 5% (23/415) for pre-diabetes to 36% (150/415) for mental health disorders (Table 3). The proportion of participants with a condition in any administrative data set ranged from 8% (34/420) for diabetes to 46% (192/420) for hyperlipidaemia (Table 4). Administrative data alone identified two to three times more cases than the questionnaire for all outcomes except hypertension and mental health disorders, where the questionnaire alone identified one to two times more cases than administrative data (Table 5). Combining all sources increased case detection for all outcomes.

**Table 3.** Number and proportion of participants with study outcomes identified from each data source.

Data sources	Questionnaire data *n*/*N*, % (95% CI)	Testsafe laboratory data set *n*/*N*, % (95% CI)	Pharmaceutical data collection *n*/*N*, % (95% CI)	National non-admitted patient collection *n*/*N*, % (95% CI)	National minimum data set *n*/*N*, % (95% CI)
Diabetes	25/415 6% (4, 8)	16/305 5% (3, 8)	28/346 8% (5, 11)	23/400 5% (3, 8)	14/411 3% (2, 5)
Pre-diabetes	23/415 5% (3, 8)	50/305 16% (12, 20)	No data	No data	No data
Total diabetes (pre-diabetes or diabetes)	48/415 11% (8, 15)	66/305 21% (17, 26)	28/346 8% (5, 11)	23/400 5% (3, 8)	14/411 3% (2, 5)
Hyperlipidaemia	135/415 32% (28, 37)	175/305 57% (51, 62)	58/346 17% (13, 21)	No data	1/411 0.2% (0.04, 1)
High blood pressure	124/415 29% (25, 34)	No data	95/346 27% (23, 32)	No data	12/411 3% (1, 5)
Mental health disorders	150/415 36% (31, 40)	No data	140/346 40% (35, 45)	No data	24/411 5% (3, 8)
Asthma	123/415 29% (25, 34)	No data	172/346 49% (44, 54)	No data	23/411 5% (3, 8)

The proportion of participants with study outcomes is defined as the ratio of participants identified with the outcome to the total number of participants who completed the questionnaire or had any records in the data source. No data outcome cannot be defined based on these data.

**Table 4.** Number and proportion of participants with study outcomes in the best informative data source and after sequentially adding administrative data sources to the best informative data source.

Outcomes	Proportion in best informative data source *n*/*N*, % (95% CI)	Proportion after adding second informative data source *n*/*N*, % (95% CI)	Proportion after adding third informative data source *n*/*N*, % (95% CI)	Proportion after adding fourth informative data source	Proportion in any administrative data sources *n*/*N*, % (95% CI)
Diabetes	28/346 8% (5, 11)	33/420 7% (5, 11)	34/420 8% (5, 11)	No addition	34/420 8% (5, 11)
	Pharmaceutical data collection	National non-admitted patient collection	Testsafe laboratory data set	National minimum data set	
Pre-diabetes	50/305 16% (12, 20)	No addition	No addition	No addition	50/420 11% (9, 15)
	Testsafe laboratory data set	No second data source	No third data source	No fourth data source	
Total diabetes	66/305 21% (17, 26)	76/420 18% (15, 22)	80/420 19 % (15, 23)	No addition	80/420 19 % (15, 23)
	Testsafe laboratory data set	Pharmaceutical data collection	National non-admitted patient collection	National minimum data set	
Hyperlipidaemia	175/305 57% (51, 62)	192/420 46% (41, 50)	No addition	No addition	192/420 46% (41, 50)
	Testsafe laboratory data set	Pharmaceutical data collection	National minimum data set	No fourth data source	
High blood pressure	95/346 27% (23, 32)	No addition	No addition	No addition	95/420 22% (18, 26)
	Pharmaceutical data collection	National Minimum data set	No third data source	No fourth data source	
Mental health disorders	140/346 40% (35, 45)	143/420 34% (29, 38)	No addition	No addition	143/420 34% (29, 38)
	Pharmaceutical data collection	National minimum data set	No third data source	No fourth data source	
Asthma	172/346 49% (44, 55)	No addition	No addition	No addition	172/420 40% (36, 45)
	Pharmaceutical data collection	National minimum data set	No third data source	No fourth data source	

The best informative data source is an administrative data source that gave the highest proportion of participants with the outcome. For each study outcome, the first row shows the proportion of participants with the outcome and the second row shows the data source. No addition means that the data source did not add any new cases. The proportion of participants with study outcomes is defined as the ratio of participants with the outcome to the total number of participants who completed the questionnaire or had records in the data source. National Health Index numbers were only available for 420 participants.

**Table 5.** Number and proportion of cases identified from each data source and agreement between them (*N* = 424), data are *n*, % (95% confidence interval).

Outcome	Cases in questionnaire data *n*, % (95% CI)	Cases in any administrative data sets *n*, % (95% CI)	Agreement between questionnaire and any administrative data set kappa (95% CI)	Cases identified by administrative data sets but not questionnaire *n*, % (95% CI)	Cases identified by questionnaire but not administrative data sets. *n*, % (95% CI)	Cases identified by both questionnaire and administrative data. *n*, % (95% CI)	Cases in any data source Questionnaire or administrative *n*, % (95% CI)	^a^Matched cohort *N* = 41,409 *n*, % (95% CI)	^b^New Zealand Health Survey 2021/2022 Age group, 45–54, % (95% CI)
Diabetes	25 5% (4, 8)	34 8% (5, 11)	0.65 (0.51, 0.80)	14 3% (1, 5)	5 1% (0.5, 2)	20 4% (3, 7)	39 9% (6, 12)	3309 7.8% (7.6, 8.1)	5.3% (3.0, 8.6)
Pre-diabetes	23 5% (3, 8)	50 11% (9, 15)	0.10 (0.02, 0.22)	44 10% (7, 13)	17 4% (2, 6)	6 1% (0.6, 3)	67 15% (12, 19)	–	–
Total diabetes (pre-diabetes or diabetes)	48 11% (8, 14)	80 18% (15, 22)	0.45 (0.34, 0.57)	46 10% (8, 14)	14 3% (1, 5)	34 8% (5, 11)	94 22% (18, 26)	–	–
Hyperlipidaemia	135 31% (27, 36)	192 45% (40, 50)	0.27 (0.18, 0.36)	103 24% (20, 28)	46 10% (8, 14)	89 20% (17, 25)	238 56% (51, 60)	4398 10.3% (10.1, 10.7)	^c^9.8% (6.8, 13.7)
High blood pressure	124 29% (25, 33)	95 22% (18, 26)	0.42 (0.32, 0.52)	33 7% (5, 10)	62 14% (11, 18)	62 14% (11, 18)	157 37% (32, 41)	7812 18.4% (18.1, 18.8)	^c^14.1% (10.5, 18.24)
Mental health disorders	^d^150 35% (30, 40)	143 33% (29, 38)	0.55 (0.46, 0.63)	40 9% (7, 12)	47 11% (8, 14)	103 24% (20, 28)	190 44% (40, 49)	13122 31% (30.5, 31.4)	Anxiety 12.1% (9.2, 15.5) Depression 22.9% (18.5, 27.7)
Asthma	123 29% (24, 33)	172 40% (36, 45)	0.44 (0.36, 0.53)	79 18% (15, 22)	30 7% (5, 9)	93 21% (18, 26)	202 47% (42, 52)	14304 33.7% (33.3, 34.2)	^c^10.6% (8.0, 13.8)

a A random population cohort matched on age, sex, and ethnicity to study participants.

b The results of the last column are from New Zealand Health Survey 2021/2022.^17^

c Diagnosed and currently taking medication.

d 65/415, 15% (12, 19) for anxiety, 132/415, 31% (27, 36) for depression.

**Figure 1.**
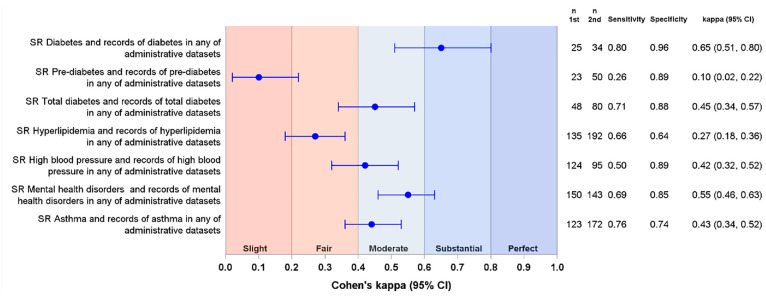
Forest plot of the agreement between questionnaire data and the records of the outcomes in any of the administrative sources (*N* = 424). Blue circles represent kappa coefficients with 95% confidence intervals (CI). In each case, second data source was compared to the first data source. SR: self-reported outcome.

**Figure 2.**
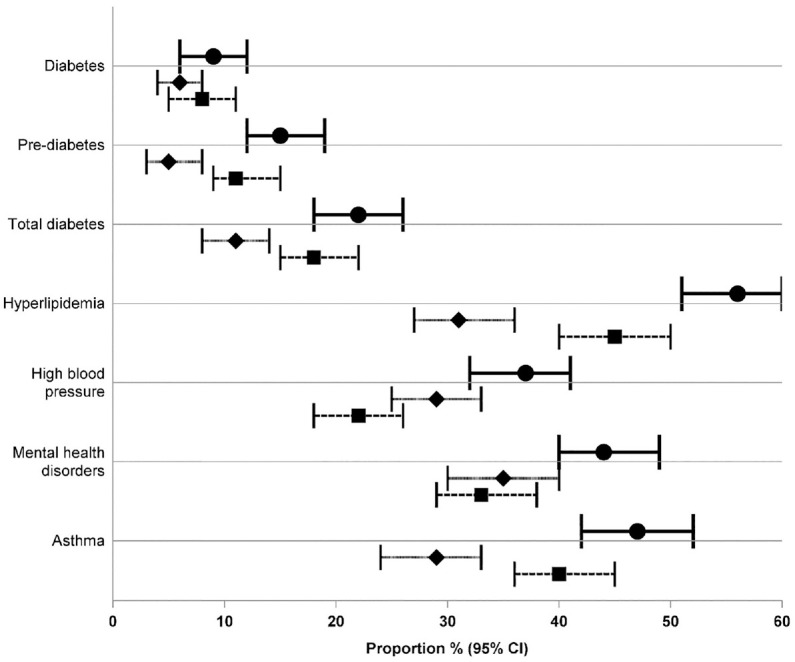
Forest plot of the proportion of each chronic condition as determined by any of the data sources, the questionnaire, and any of the administrative data sources. Circle and solid line, any data source; diamond and short dash, questionnaire data; square and long dash, any of the administrative data sets.

Agreement between the questionnaire and administrative data was substantial for diabetes (kappa = 0.65, 95% CI = 0.50, 0.80), slight for pre-diabetes (kappa = 0.10, 95% CI = 0.02, 0.22), fair for hyperlipidaemia (kappa = 0.27, 95% CI = 0.18, 0.36), and moderate for total diabetes, high blood pressure, mental health disorders, and asthma (Figure 1). The probability of the administrative data sources identifying self-reported conditions ranged from 26% for pre-diabetes to 80% for diabetes. The specificity for excluding self-reported conditions ranged from 64% for hyperlipidaemia to 96% for diabetes (Figure 1). When the proportion of participants with each outcome was estimated from questionnaire data, any of the administrative data sets, and any data source, the confidence intervals showed substantial overlap for diabetes and mental health disorders but minimal or no overlap for other outcomes (Figure 2).

To determine the relative contribution of each of the administrative data sets in identifying the outcomes, we sought the best informative data source that is the data source that identified the most cases. The pharmaceutical collection and the Testsafe laboratory data set identified the highest number of cases (Table 4). Adding other data sources to the best informative data sources added few additional cases (Table 4).

### Validation of questionnaire and administrative data

#### Expert panel

There was substantial or perfect agreement between questionnaire and administrative data and the expert panel for all outcomes except pre-diabetes, which had fair agreement for questionnaire data (Table 6). There was also perfect agreement (all kappa >0.89) between expert panel and any data source for all outcomes. Expert panel review resulted in a decrease in the number of participants with each outcome of interest, with the biggest decreases in the proportion with diabetes (7/39 cases identified by any data source not confirmed, or 18%), pre-diabetes (9/67, 13%), and asthma (22/202, 11%). Most cases not confirmed by the expert panel had records of the outcome of interest in only one data set and were judged to have received treatment for other reasons, not related to the outcome of interest (Table 6).

**Table 6.** Number and proportion of cases identified by each data source and agreement with expert panel.

Outcomes	Identified by questionnaire and confirmed by expert panel *n*, *N* % (95% CI)	Identified by questionnaire, but not confirmed by expert panel *n*, *N* % (95% CI)	Agreement between expert panel review and questionnaire data kappa (95% CI)	Identified by administrative data sets and confirmed by expert panel *n*, *N* % (95% CI)	Identified by administrative data sets, but not confirmed by expert panel *n*, *N* % (95% CI)	Agreement between expert panel review and administrative data kappa (95% CI)	Identified by any data sources and confirmed by expert panel *n*, *N* % (95% CI)	Identified by any data sources, but not confirmed by expert panel *n*, *N* % (95% CI)	Agreement between expert panel review and any data sources kappa (95% CI)
Diabetes	24/415 5% (3, 8)	1/415 0.2% (0.04, 1)	0.83 (0.72, 0.94)	28/420 6% (4, 9)	6/420 1% (0.6, 3)	0.84 (0.74, 0.94)	32/424 7% (5, 10)	7/424 1% (0.4, 2)	0.89 (0.81, 0.97)
Pre-diabetes	18/415 4% (2, 6)	5/415 1% (0.5, 2)	0.40 (0.26, 0.53)	46/420 10% (8, 14)	4/420 0.9% (0.03, 2)	0.83 (0.75, 0.91)	58/424 13% (10, 17)	9/424 2% (0.7, 3)	0.92 (0.86, 0.97)
Total diabetes (pre-diabetes or diabetes)	47/415 11% (8, 14)	1/415 0.2% (0.04, 1)	0.63 (0.53, 0.72)	77/420 18% (14, 22)	3/420 0.07% (0.02, 2)	0.88 (0.83, 0.94)	90/424 21% (17, 25)	4/424 0.9% (0.3, 2)	0.97 (0.95, 1.00)
High blood pressure	124/415 29% (25, 34)	0	0.86 (0.80, 0.91)	89/420 21% (17, 25)	6/420 1% (0.6, 3)	0.62 (0.54, 0.70)	151/424 35% (31, 40)	6/424 1% (0.6, 3)	0.97 (0.95, 0.97)
Asthma	123/415 29% (25, 34)	0	0.71 (0.65, 0.78)	150/420 35% (31, 40)	22/420 5% (3, 7)	0.75 (0.68, 0.81)	180/424 42% (37, 47)	22/424 5% (3, 7)	0.90 (0.85, 0.94)

Expert panel review was the gold standard. The expert panel reviewed participants for whom there were discrepancies between the questionnaire and the administrative data or if there was evidence from only one administrative data set (*n* = 43 for diabetes or pre-diabetes, 14 for high blood pressure, and 44 for asthma). Numbers presented in the table include those reviewed by the panel plus those considered to have the condition because there was evidence from more than one source. National Health Index numbers were only available for 420 participants.

#### Virtual diabetes register

The proportion of participants with diabetes in the virtual diabetes register was 6% (29/424, 95% CI = 4, 9). The virtual diabetes register identified 28 of 39 cases identified by any data source. The virtual diabetes register showed substantial agreement (kappa = 0.68, 95% CI = 0.54, 0.83) with self-reported diabetes mellitus, and perfect agreement with records of diabetes in administrative data sets (kappa = 0.88, 95% CI = 0.79, 0.97) as well as with records of diabetes in any data source (kappa = 0.81, 95% CI = 0.70, 0.91).

#### New Zealand Health Survey

The proportion of chronic conditions in the questionnaire data was higher than that in the New Zealand Health Survey for the age group of 45–54 years for all outcomes except diabetes, where the prevalence was almost 5% in both (Table 5).

#### Integrated data initiative

The integrated data infrastructure matched cohort (*n* = 42,400) were born from December 1969 to February 1974 and alive in July 2023, and matched study participants on sex and ethnicity. The proportion with the chronic conditions of interest in any data source was higher than that in the matched cohort for all outcomes except diabetes, where the prevalence was almost 9% in both (Table 5).

## Discussion

We aimed to determine whether self-reported questionnaires can be replaced with administrative data sets to identify chronic conditions for follow-up of participants in a randomized trial conducted in New Zealand. We found that these data sources are not interchangeable, and the outcomes would be underestimated using either of these sources alone. This study suggests the two data sources are additive. Use of both sources could increase case ascertainment and thus potentially increase power for detection of differences between randomized groups.

We found that a combination of self-reported questionnaire, the Pharmaceutical collection, and the Testsafe laboratory data set were sufficient to identify almost all participants with the outcomes of interest. Thus, for future studies, these three sources are likely to be sufficient for identifying these chronic conditions in a cohort with a similar sample frame.

We chose the questionnaire data as the initial gold standard, as some participants were living overseas and self-report may be the only way to obtain their data. Several studies investigating the validity and reliability of self-reported data have reported high accuracy and reliability compared with administrative data sets.^30,31^ Others have also reported substantial agreement between self-reported questionnaire data and administrative data sets.^32–35^

However, we found a low level of agreement between data sources for these chronic conditions. This may be in part because for some outcomes, particularly pre-diabetes and hyperlipidaemia, participants may not be aware of their laboratory results. For instance, individuals with these chronic conditions usually manage them initially through lifestyle modifications, such as diet and physical activity. Some may be less engaged in their health care, and rely only on guidance from their health care provider. As a result, they may not self-report having the condition.

In contrast, agreement between data sources was high for diabetes. This could be because diabetes requires more medical attention and monitoring than other outcomes in our study. With frequent contact with health care services, and early initiation of medication, patients with diabetes are more likely to have their medical records captured in different data sources and also gain better awareness of their condition. However, for hyperlipidaemia, initiation of lipid-lowering medication is usually based on an individual’s blood lipid profile in the context of their cardiovascular risk, rather than solely an elevated cholesterol concentration. Therefore, prescriptions for hyperlipidaemia were only recorded for some participants, even when their laboratory results indicated the condition.

We undertook several validation steps to assess the overall validity of our conclusions about prevalence of the outcomes in our study cohort. The expert panel review showed the prevalences were likely overestimated by any data source, especially for diabetes, pre-diabetes, and asthma. In most cases this involved individuals who had received treatment for reasons unrelated to the outcome of interest. For example, a small number of participants had metformin prescriptions alone with no laboratory data to confirm a diagnosis of diabetes. Our expert panel ultimately classified them as not having diabetes. In addition, inhaled respiratory medications, such as bronchodilators, which are commonly used to treat asthma, could also be used for other respiratory conditions. Since the criteria we used were intended to have high sensitivity, and these multipurpose pharmaceuticals have lower specificity, expert panel review and/or more stringent criteria in combining administrative data could be used address this issue. The overestimation of these outcomes using the combined data sets could result in bias towards the null.^36,37^ To mitigate overestimation in future studies, participants with records in only one data set or regionally limited data may need adjudication.

The virtual diabetes register identified 71% of the diabetes cases identified from the combined data sources. The virtual diabetes register is not intended for individual identification of diabetes cases; rather, it is designed to estimate the prevalence of diabetes in the population.^38^ The virtual diabetes register algorithm does not utilize the actual results of HbA1c tests.^38^ Moreover, the virtual diabetes register could not identify individuals living overseas. A previous study reported the virtual diabetes register identified 87% of diabetes cases identified from Testsafe laboratory data.^39^ The poorer case ascertainment by the virtual diabetes register compared with our study may be because we included cases identified from additional sources, including questionnaire data. However, there was substantial overlap in the confidence intervals for the proportion of participants with diabetes estimated from the virtual diabetes register and from the combined data sources.

The proportion of participants with self-reported chronic conditions in our study was higher than that in the New Zealand Health Survey for all outcomes except diabetes, where the proportion was similar. This might be because our study was investigating the risk factors for cardiovascular disease, so we asked participants if they had ever been diagnosed by a doctor as well as what treatments they received. However, the survey asked this question only for diabetes, whereas for hyperlipidaemia, high blood pressure, and asthma, respondents were only asked if they were taking medication. Since these conditions are often untreated, this would have resulted in a lower proportion of those conditions.

The proportion of participants with chronic conditions identified from the combined data sources, or from the administrative data alone, was higher than that in the integrated data infrastructure cohort for all outcomes except diabetes, where it was similar. However, 71% of our cohort were born preterm, whereas in New Zealand from 2012 to 2021, the preterm birth rate ranged from 7.6% to 7.9%,^40^ and was <5% in 1998.^41^ Preterm birth is independently associated with increased risk of chronic disease outcomes,^42,43^ and this may have contributed to the higher proportion of our cohort with the outcomes of interest than the integrated data infrastructure cohort.

Although Cohen’s kappa is subject to a variety of limitations, we have chosen to present kappa throughout this manuscript for consistency and accessibility, as it is widely understood, and its established categorization contributes to interpretation. However, the kappa statistic may appear paradoxical in cases of high prevalence with correction for the amount of agreement by chance alone. The kappa coefficient calculated when prevalence exceeds 60% may appear biased.^44^ Gwet’s AC1 agreement statistic is not influenced by prevalence in this way. We recalculated agreement statistics using Gwet’s AC1 statistic. Both higher and lower kappa and Gwet AC1 statistics agreed for common conditions with higher prevalence, such as mental health disorders and hyperlipidaemia, as well as for uncommon conditions like diabetes and pre-diabetes. For example, only hyperlipidaemia approached 60% prevalence, and kappa and AC1 were similar (kappa = 0.27 (95% CI = 0.18, 0.36) AC1 = 0.21 (95% CI = 0.10, 0.33).

Although this study relates to the New Zealand setting, the findings are potentially useful for researchers from other countries who wish to determine trial outcomes in a similar way. Many countries have a nationwide repository of clinical data that encompass most of a patient’s clinical care. Second, data linkage requires a unique identifier that can be linked to medical data. New Zealand has the national health index number, but if a unique identifier is not available, probabilistic matching is possible with name, sex, date of birth, and other factors to link data sets. Third, robust medical ethics and data sharing/availability processes are required. Although these are in place in New Zealand, accessing administrative data has required a long period of time for the application and data extraction processes.^45^

### Strengths of the study

Our study has some strengths. First, in New Zealand, record linkage is possible via the National Health Index, a unique identifier attached to every participant in the health system. Another strength is that New Zealand has established data collection protocols and coding, which help ensure that participants’ records are collected in a standardized manner with appropriate governance procedures. Finally, we obtained a high rate of consent to access all data sources.

### Limitations of the study

Our study has some limitations. First, our study had no in-person clinical assessment to act as a gold standard for comparisons. Second, the study was limited to a relatively small number of similarly aged participants, and thus our findings may not be generalizable to participants of different ages where the prevalence of chronic diseases may differ. In addition, we recruited offspring of participants in a randomized trial. Since those who participate in such trials may not be representative of the population, this may limit the generalizability of the findings. Third, we had no access to primary care records where blood pressure and primary care physician diagnoses would be recorded. Privately funded health care contacts also may not have been included in the administrative data sources. Finally, the administrative data did not include records of participants living overseas, nor did we have access to laboratory data collected outside the Northern region. These last two limitations could reduce the robustness of our findings and may underestimate the number of individuals with specific outcomes, although our comparison with the validation data sets suggests that underestimation is unlikely to be substantial.

Our validation attempts also have limitations. First, the expert panel only had access to the questionnaire and administrative data but not primary care records, though they viewed the totality of those data. Second, the integrated data infrastructure cohort could not match for the high proportion of preterm births in the inception cohort.

## Conclusion

We aimed to explore whether self-reported questionnaire data can be used interchangeably with data from administrative data sets to identify chronic conditions in the follow-up of participants in a randomized trial. We found that self-reported questionnaire data cannot be replaced with administrative data, as they each identified additional cases for all outcomes of interest. The pharmaceutical collection and laboratory results from Testsafe, in combination with questionnaire data, maybe sufficient to identify chronic diseases, although adjudication should be considered for participants with records of the condition in only one data source.

## Supplemental Material

sj-docx-1-ctj-10.1177_17407745241259088 – Supplemental material for Comparison of outcomes of the 50-year follow-up of a randomized trial assessed by study questionnaire and by data linkage: The CONCUR study Supplemental material, sj-docx-1-ctj-10.1177_17407745241259088 for Comparison of outcomes of the 50-year follow-up of a randomized trial assessed by study questionnaire and by data linkage: The CONCUR study by Mohammad Shahbaz, Jane E Harding, Barry Milne, Anthony Walters, Lisa Underwood, Martin von Randow, Lois Xu and Greg D Gamble in Clinical Trials
