# Supplementary material for: Comparison of outcomes of the 50-year follow-up of a randomized trial assessed by study questionnaire and by data linkage: The CONCUR study
Source: Clin Trials. 2024 Jun 22;22(1):24–35. doi: 10.1177/17407745241259088 (PMC11809116; doi:10.1177/17407745241259088)
Supplement: sj-docx-1-ctj-10.1177_17407745241259088 – Supplemental material for Comparison of outcomes of the 50-year follow-up of a randomized trial assessed by study questionnaire and by data linkage: The CONCUR study [file sj-docx-1-ctj-10.1177_17407745241259088.docx]

**Supplementary table 1.** Questions regarding study outcomes from self-reported questionnaire.

| **Outcome** | **Question** |
| --- | --- |
| Diabetes, pre-diabetes, and total diabetes | Have you ever been told by a doctor that you have pre-diabetes or diabetes? (not including gestational diabetes)/ What type of diabetes have you been told that you have? (pre-diabetes, type 2 diabetes, type 1 diabetes, other) |
| Hyperlipidemia | Have you ever been told by a doctor that you have high blood cholesterol levels? (Yes, No, Don’t know) |
| High blood pressure | Have you ever been told by a doctor that you have high blood pressure? (Yes, No, Don’t know) |
| Mental health disorders | Have you ever been told by a doctor that you have depression? (Yes, No, Don’t know) |
|  | Have you ever been told by a doctor that you have anxiety disorder? (Yes, No, Don’t know) |
| Asthma | Have you ever been told by a doctor that you have asthma? (Yes, No, Don’t know) |

**Supplementary table 2.** Datasets included in the integrated data infrastructure with inclusion dates.

| **Dataset** | **Availability of data** |
| --- | --- |
| Cancer registrations (Cancer Register) | 1995 to December 2021 |
| Chronic conditions table (IDI summary table) (MoH CC)^a^ | 1985 to December 2017 |
| Hospital discharges public (NMDS) | 1988 to June 2022 |
| Pharmaceutical data | 2005 to December 2022 |
| Mental health contacts (PRIMHD) | 2008 to June 2022 |
| Outpatient visits and short hospital stays (NNPAC) | 2007 to June 2022 |
| Laboratory claims | 2003 to December 2020 |
